# CD38: A Significant Regulator of Macrophage Function

**DOI:** 10.3389/fonc.2022.775649

**Published:** 2022-02-17

**Authors:** Wentao Li, Yanling Li, Xi Jin, Qianjin Liao, Zhifang Chen, Honghua Peng, Yanhong Zhou

**Affiliations:** ^1^ NHC Key Laboratory of Carcinogenesis and Hunan Key Laboratory of Cancer Metabolism, Hunan Cancer Hospital and the Affiliated Cancer Hospital of Xiangya School of Medicine, Central South University, Changsha, China; ^2^ Cancer Research Institute, Basic School of Medicine, Central South University, Changsha, China; ^3^ Department of Nuclear Medicine, Hunan Cancer Hospital and The Affiliated Cancer Hospital of Xiangya School of Medicine, Central South University, Changsha, China; ^4^ Department of Gynecology, The First Affiliated Hospital of Xinjiang Medical University, Urumqi, China; ^5^ Department of The Oncology, Third Xianya Hospital, Xiangya School of Medicine, Central South University, Changsha, China

**Keywords:** CD38, macrophages, calcium, SASP (senescence-associated secretory phenotype), mechanism

## Abstract

Cluster of differentiation 38 (CD38) is a cell surface glycoprotein and multifunctional extracellular enzyme. As a NADase, CD38 produces adenosine through the adenosine energy pathway to cause immunosuppression. As a cell surface receptor, CD38 is necessary for immune cell activation and proliferation. The aggregation and polarization of macrophages are affected by the knockout of *CD38*. Intracellular NAD^+^ levels are reduced by nuclear receptor liver X receptor-alpha (LXR) agonists in a CD38-dependent manner, thereby reducing the infection of macrophages. Previous studies suggested that CD38 plays an important role in the regulation of macrophage function. Therefore, as a new marker of macrophages, the effect of CD38 on macrophage proliferation, polarization and function; its possible mechanism; the relationship between the expression level of CD38 on macrophage surfaces and disease diagnosis, treatment, etc; and the role of targeting CD38 in macrophage-related diseases are reviewed in this paper to provide a theoretical basis for a comprehensive understanding of the relationship between CD38 and macrophages.

## Introduction

Cluster of differentiation 38 (CD38) is a cell surface glycoprotein and multifunctional extracellular enzyme which is primarily a NAD^+^ glycohydrolase and ADPR cyclase (1). As a nicotinamide adenine dinucleotide (NAD^+^) hydrolase, most of the NAD^+^ catalyzed by CD38 is converted to adenosine diphosphate-ribose (ADPR), and a few molecules are cyclized to form cADPR by ADPR cyclase (2). At an acidic pH, CD38 catalyzes the synthesis of nicotinic acid adenine dinucleotide phosphate (NAADP) from NADP^+^ (3). cADPR and NAADP are secondary messengers involved in calcium regulation and mobilization, such as calcium signal transduction and release, leading to angiogenesis and tumor progression (4–6). In addition, ADPR is further processed (through CD203a and CD73) to form ADO to cause immunosuppression (2).

As a cell surface receptor, CD38 is necessary for immune cell activation and proliferation. High expression of CD38 in immune cells can affect their functions, such as in macrophages, regulatory T cells (Tregs), regulatory B cells (Bregs), myeloid-derived suppressor cells (MDSCs) and CD16^-^CD56^+^ natural killer (NK) cells (7). Macrophages are usually divided into M1 and M2 subsets, which have different functions and transcriptional profiles. The balanced polarization of M1 and M2 macrophages determines the fate of an organ during inflammation or injury (8–10). Pro-inflammatory M1-like macrophages accumulate in metabolic tissues during aging and the acute inflammatory response, including visceral white adipose tissue and the liver. These M1-like macrophages express high levels of the NAD consuming enzyme CD38 and enhance CD38-dependent NADase activity, thereby reducing the tissue NAD^+^ level. A study also found that aging cells gradually accumulated in the visceral white adipose tissue and liver during aging, and the inflammatory cytokines secreted by aging cells (representing the senescence-associated secretory phenotype, SASP) induced macrophage proliferation and the expression of CD38 (11). *In vitro*, it was found that lipopolysaccharide (LPS) in macrophages could upregulate the expression of CD38 in a time-and-dose-dependent manner. Knocking out or blocking CD38 inhibited LPS-induced M1 polarization of macrophages (12). Thus, CD38 is an important regulator of macrophage function. The present review discusses the effects of CD38 expression on the proliferation and polarization of macrophages, the effect of CD38 on macrophage function and its possible mechanisms, the expression level of CD38 on macrophage surfaces related to disease diagnosis, treatment, and prognosis; and the role of targeted CD38 therapy in macrophage-related diseases. Thus, this review aimed to promote our understanding of the relationship between CD38 and macrophages.

## CD38 Is a Potential New Marker of Macrophages

CD38 was initially considered as a surface activation marker of T cells and was later found to be expressed in other immune and non-immune cells, including macrophages (5). Under appropriate stimulation, macrophages are activated into an inflammatory state, which can be divided into M1 (classical activation) and M2 (alternative activation) subsets. The former are immune effector cells that resist bacterial invasion and phagocytizes and digest necrotic cells, and the latter are mainly responsible for wound healing and tissue repair. The M2b subtype of M2 macrophages has strong immunomodulatory and anti-inflammatory effects (13, 14). Through the comprehensive analysis of the transcriptional characteristics of M1 and M2 macrophages of mice, Jablonski et al. found that CD38 is upregulated by more than 50 times in M1 macrophages, while its expression is downregulated in M2 macrophages compared with that in M0 macrophages, which suggests that CD38 is the exclusive expression pattern of M1 macrophages, while early growth response protein 2 (EGR2) is the exclusive expression pattern of M2 macrophages. Further verification by flow cytometry showed that M1 and M2 macrophages could be distinguished by their relative expression levels of CD38 and EGR2. CD38 labeled most (71%) M1 macrophages *in vitro* (14). However, some studies have shown that bone marrow-derived macrophages (BMDMs) not infected with *Coxiella burnetii*-nine-mile phase II (NMII) strain do not significantly express CD38 and EGR2, the markers of classical activated (M1) and alternative activated (M2) macrophages (15).

M1 macrophages are usually activated by LPS and interferon gama (IFN-γ). After activation, they produce a large amount of interleukin 12 (IL-12) and a small amount of IL-10. Amici et al. found that CD38 is a marker of inflammatory macrophages *in vitro* and in an *in vivo* mouse model. Monocyte analysis in patients with systemic lupus erythematosus showed that although all monocytes expressed CD38, high expression of CD38 in atypical monocyte subsets was related to the disease. These data are consistent with the inflammatory marker role of CD38 in human macrophages and monocytes (9). In addition, Jablonski et al. also found that CD38 was selectively upregulated in BMDMs of inflamed mice (14). *Mycobacterium tuberculosis* cell wall binding factor, trehalose 6,6’-dimethylcholic acid (TDM), is a physiologically useful molecule that can be used to establish macrophage-mediated early events in the pathogenesis of tuberculous granuloma. Seven days after TDM introduction, CD11b^+^CD45^+^ macrophages with high surface expression of M1 (inflammatory)-like markers CD38 and CD86 were found in the pathological areas of mouse lung tissues (16). In summary, CD38 can be used as a potential new marker of macrophages.

## Effect of CD38 on Macrophage Proliferation and Polarization

Macrophages exert an effector function against invading microorganisms. CD38 plays an important role in the proliferation and polarization of various immune cells, including macrophages (7). Knockout of *CD38* affected the aggregation and polarization of macrophages. Studies have shown that LPS can induce a series of inflammatory reactions and signal transductions. In cultured macrophages, LPS could upregulate the level of CD38 in a time and dose-dependent manner. It also increased the expression of *CD38* at the mRNA level by activating the Janus kinase signal transducer and transcription activator (JAK-STAT) pathway (17). However, knocking out or blocking CD38 in macrophages might inhibit the M1 polarization of macrophages induced by LPS and reduce nuclear factor kappa B (NF-κB) signal activation. CD38 can regulate the macrophage activation and acute renal injury (AKI) caused by LPS, and can be used as a therapeutic target for AKI caused by sepsis (12).

The recovery of CD38-deficient mice after closed head injury (CHI) was significantly lower than that of wild-type (WT) mice, and the object recognition ability of CD38-deficient mice was significantly lower than that of the WT mice. In addition, we observed that the number of activated microglia/macrophages at the injury site in the CD38-deficient mice was significantly lower than that in the WT mice, while CD38 was expressed in brain microglia. Thus, CD38 plays a beneficial role in the recovery of CHI of mice, and this effect is mediated, at least in part, by an increase in the number of microglia/macrophages, mediated by CD38 (18). It can be seen that CD38 is conducive to macrophage proliferation and polarization.

## Effect of CD38 on Macrophage Function and Its Possible Mechanisms

### Effect of CD38 on the Intracellular Calcium Concentration of Macrophages

CD38 is a regulator of the intracellular calcium pool and has the characteristics of anti-osteoclastogenesis. Increased expression of CD38 can reduce the number of osteoclasts and bone resorption. Through Ca^2+^, cAMP, and cytokines (such as tumor necrosis factor alpha (TNF-α), regulating the expression of the NAD^+^ sensitive enzyme CD38 might help to couple the strong metabolic activity of osteoclasts and osteoblasts with their respective bone resorption and bone shaping functions (19, 20). In the tumor microenvironment (TME), CD38 regulates the activation of tumor associated microglia/macrophages (TMMs) through the increase of calcium concentration mediated by cADPR. TMMs contribute to an immunosuppressive TME, thus promoting the growth and metastasis of glioma. In addition, the inflammatory process can significantly affect the brain injury resulting from ischemic stroke. The synthesis of calcium by bifunctional receptors and extracellular enzyme CD38 mobilizes the increased calcium concentration mediated by second messengers (e.g., cADPR), which has been proven to be necessary for the activation and migration of myeloid immune cells. The activation, migration, and accumulation of immune cells are the key steps in post-ischemic inflammation. Cu et al. found that CD38 deficiency could reduce the production of chemokines, immune cell infiltration (macrophages and lymphocytes) and brain injury after transient ischemia-reperfusion. Therefore, CD38 might be a therapeutic target for ischemic stroke (21).

### Effect of CD38 on the Phagocytosis of Macrophages

Macrophages have a powerful effector function against invading microorganisms. Macrophages internalize pathogens through phagocytosis, then kill them and digest them in phagosomes (22). CD38 can reduce the risk of infection of macrophages. Matalonga identified a molecular mechanism regulated by the nuclear receptor liver X receptor-alpha (LXR), which limits the infection of host macrophages through the transcriptional activation of multifunctional enzyme CD38 (22). LXR agonists reduce intracellular NAD^+^ levels in a CD38-dependent manner, resist pathogen-induced changes in macrophage morphology and the F-actin cytoskeleton distribution, and reduce the ability of non-conditioned *Salmonella* to infect macrophages (22). NAD^+^ supplementation reversed the morphological transformation of macrophages and the accumulation of dorsal F-actin, and restored the ability of *Salmonella* to infect macrophages in the presence of LXR agonists. Therefore, the mechanism comprises limiting the infection of host macrophages by affecting CD38 and regulating NAD^+^ metabolism.

In addition, CD38^+^ macrophages can limit the growth of intracellular bacteria. Pathogens such as *Haemophilus aegypt*i, *Haemophilus influenzae*, *Haemophilus haemolyticus*, *Haemophilus parainfluenzae*, and *Haemophilus parahaemolyticus* lack the ability to synthesize NAD^+^; therefore, they rely on the uptake of NAD^+^ and NAD^+^ precursors [e.g., nicotinamide mononucleotide (NMN) and nicotinamide ribose (NR)] to support metabolism and growth. In fact, NAD^+^ and its precursors are necessary for bacterial growth and must be included in the culture medium as factor V. CD38 exists in activated immune cells. As an extracellular enzyme or intracellular enzyme, CD38 promotes the metabolic collapse of pathogens by degrading NAD^+^ and its precursors, thus limiting the development or progress of infection. This occurs not only outside the cell, but also inside the cell (23).

### CD38 Is Involved in Nerve Injury and Protection of Macrophages

After facial nerve transection in *Cd38* knockout mice, axonal degeneration and demyelination were delayed, and macrophage aggregation decreased. Supplementation with NAD^+^ could slow down axonal degeneration and demyelination, but did not change the level of macrophage infiltration after amputation. CD38 deletion and NAD^+^ supplementation may have an autonomic protective effect on axonal cells after facial nerve transection (24). Normal or pathological aging is characterized by an increase in the number of aging cells in the brain, mainly astrocytes, which display SASP and are characterized by the release of pro-inflammatory cytokines and chemokines. These pro-inflammatory factors increase the expression of CD38 in astrocytes and microglia (resident brain macrophages), resulting in (i) increased release of pro-inflammatory cytokines and neuroinflammation, and (ii) NAD depletion, accumulation of DNA damage, metabolic dysfunction, and oxidative stress caused by the decreased activity of NAD-dependent enzymes, such as sirtuins and poly(ADP-ribose) polymerase (PARP), resulting from increased CD38 enzyme activity, leading to neuronal damage and eventually, cell death (25).

### The Change of CD38 Expression on Macrophage Is Involved in the Regulation of NAD^+^ Level

**Figure 1:**
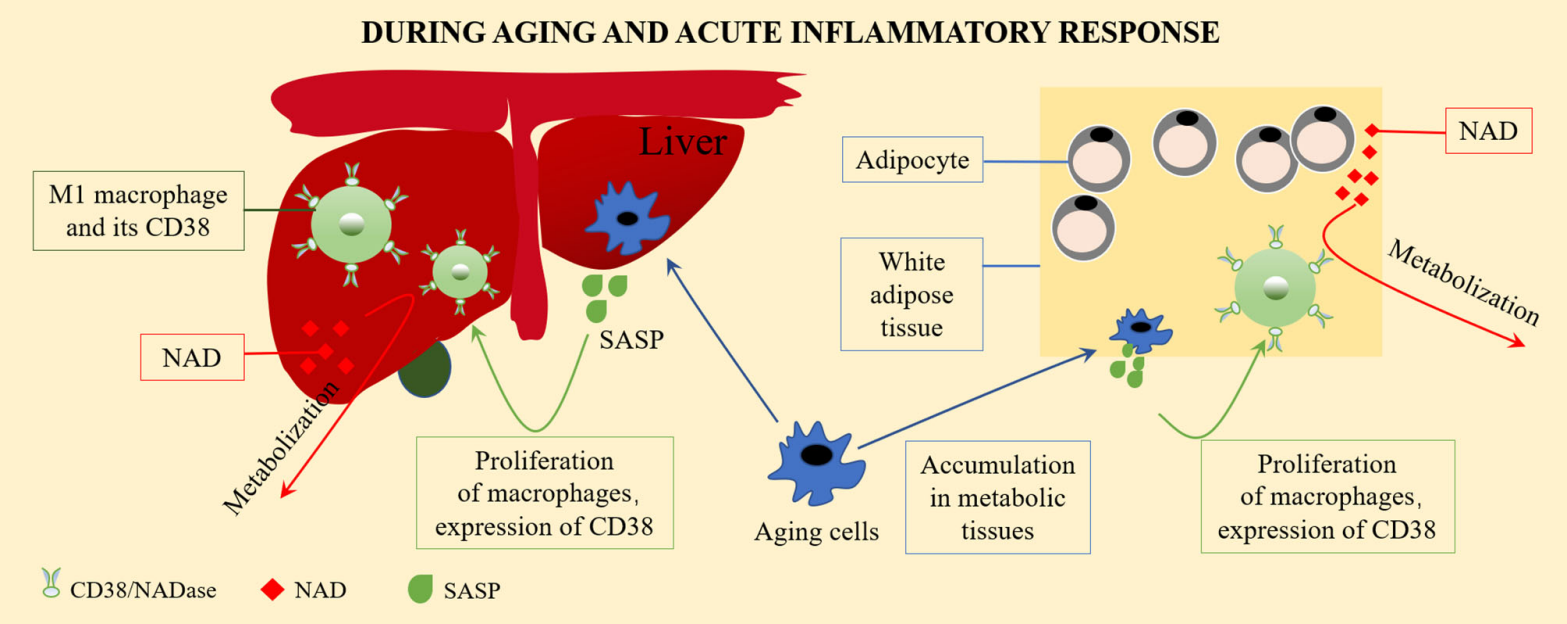
The CD38 expression on macrophage and the regulation of NAD^+^ level. Aging cells gradually accumulate in visceral white adipose tissue and liver during aging. Inflammatory cytokines secreted by aging cells (senescence associated secretory phenotype, SASP) induce macrophages to proliferate and to express CD38. Pro-inflammatory M1-like macrophages accumulate in metabolic tissues, including visceral white adipose tissue and the liver, during aging and acute inflammatory responses. These M1-like macrophages express high levels of the NAD-consuming enzyme CD38 and enhanced CD38-dependent NADase activity, thus reduces tissue NAD levels.

In many tissue types, the expression of CD38 increases with aging. As a NAD^+^ hydrolase, CD38 significantly downregulates the level of NAD^+^. During aging, the activation of CD38 might increase NF-κB signal transduction. The reason for this is that NF-κB of *Cd38* KO mice was greatly weakened in a collagen-induced arthritis mouse (CIA) model (26). The NF-κB signaling pathway is one of the main signaling pathways involved in the emergence of SASP (27), and most pro-inflammatory genes expressed in aging cells are related to NF-κB (28). The activation of tissue resident macrophages in SASP and the inflammatory environment causes the accumulation of macrophages in the liver, which expresses more CD38, showing increased signs of aging, and tending to promote inflammatory polarization (24). Covarrubias et al. found that aging cells gradually accumulated in the visceral white adipose tissue and liver during aging. Inflammatory cytokines secreted by aging (SASP) cells induced macrophage proliferation and expressed CD38. In addition, pro-inflammatory M1-like macrophages, rather than naive or M2 macrophages, were accumulated in metabolic tissues, including the visceral white adipose tissue and liver, during aging and the acute inflammatory response. These M1-like macrophages expressed high levels of the NAD-consuming enzyme CD38 and enhanced CD38-dependent NADase activity, thereby reducing the tissue NAD level (11), as described in **Figure 1**. The CD38 expression of macrophages might be the main reason for the decline of NAD^+^ in aging tissues.

In the kidney, lung, blood vessels, brain, and other important human tissues, the number of aging cells related to injury or stress secreting pro-inflammatory factors exhibiting SASP increases with age. SASP-conditioned mediators from aging cells can also induce the expression of CD38 in macrophages and endothelial cells. These M1-like macrophages express a high level of CD38 and enhance the CD38 dependent NADase activity, thus reducing the tissue NAD level, while the decrease in NAD^+^ levels related to aging might weaken SASP and reduce its pathological effect (29–31).

## The Relationship Between the Expression Level of CD38 on the Macrophage Surface and Disease Diagnosis, Treatment, and Prognosis

The increase of macrophage CD38 is observed in many diseases, for example, the expression of renal macrophage CD38 in lupus nephritis is significantly increased (32). The cell surface CD38 expressed by small macrophages was significantly increased in lung resection tissues from patients with chronic obstructive pulmonary disease (COPD) patients (33). Among 54 patients infected with HIV with virus inhibition, the level of CD14CD38 (% CD14) increased significantly in 20 patients with significantly reduced memory ability (34). This suggested that the expression level of CD38 on the surface of macrophages might be related to the diagnosis of disease.

In the tumor microenvironment, CD38^+^ macrophages contribute to the anti-tumor effect. For example, the density of CD38^+^ CD68^+^ macrophages is associated with the improvement of postoperative prognosis of liver cancer. The density of CD68^+^ macrophages is associated with poor prognosis, while CD38 is significantly expressed in the internal environment of human macrophages, which produces high levels of IL-6 and TNF-α, and together with the expression of CD80, it causes more inflammation and helps to inhibit the tumor. Therefore, the density of CD38^+^ macrophages might correlate positively with the prognosis of patients with hepatocellular carcinoma (HCC) and might be meaningful for routine diagnosis (1, 3, 35). Thus, it can be seen that the expression level of CD38 on the surface of macrophages might be associated with the prognosis of disease.

Tumor associated microglia/macrophages (TMMs) show different or even opposite effects to CD38^+^ macrophages. TMMs are formed by a small number of CD38^+^ microglia and infiltrating monocytes in the brain. They secrete IL-1, basic fibroblast growth factor, and VEGF, and regulate Ca^2+^ mobilization through CD38-mediated cADPR, which is conducive to TMM activation, angiogenesis, and immunosuppression (1), thus promoting the growth and metastasis of glioma. Therefore, the expression of CD38 in TMMs might correlate negatively with the prognosis of patients with glioma. Subsequent related studies also supported this view. For example, compared with wild-type mice carrying glioma, CD38-deficient mice showed reduced expansion of glioma and prolonged life. Similar results were obtained by targeting CD38 pharmacologically by administering the CD38 inhibitor, k-rhein (2). The mechanism comprises the activation of immune cells (including T cells, natural killer cells, neutrophils, macrophages, and dendritic cells) by inhibiting the formation of adenosine by inhibiting the NAD glycohydrolase activity of CD38. By contrast, through the inhibition of CD38, adenosine further inhibits the antitumor immune response under hypoxia by recruiting bone marrow-derived inhibitory cells and T regulatory cells, which enhance the activity of T effector cells and limit tumor progression (36, 37). This shows that inhibiting CD38 in patients’ glioma is conducive to the treatment of glioma and prolong life.

## The Role of Targeted CD38 (Therapy) in Diseases

### The Role of an Anti-CD38 Monoclonal Antibody (mAb)and Its Synergy in the Treatment of Multiple Myeloma

**Figure 2:**
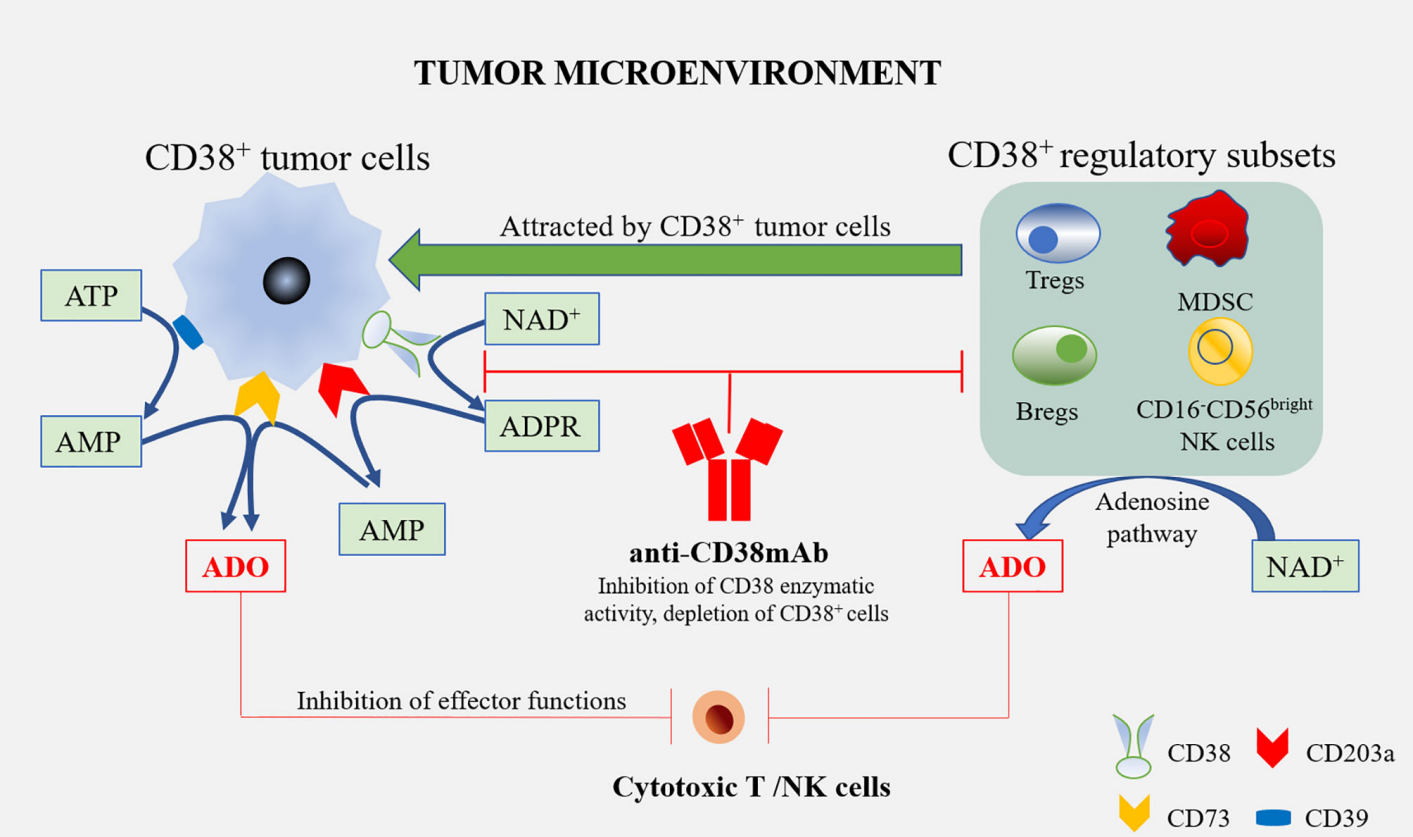
Anti-CD38 mAbs and the treatment of multiple myeloma. Tumor cells are able to attract diverse regulatory subsets, such as Tregs, Bregs, MDSCs, and CD16^-^CD56^bright^ NK cells. All these cells may co-operate in the production of ADO, starting from ATP or NAD^+^. ADO produced by these cells, as well as tumor cells in the tumor microenvironment, interact with ADO receptors on the cytotoxic T/NK cells to inhibit effector functions, which alleviates the anti-tumor immune response. The treatment of multiple myeloma using Anti-CD38 mAbs might overcome the immune suppression by blocking CD38’s enzymatic activity in these cells, and the depletion of CD38^+^ cells leads to an increased therapeutic response.

Daratumumab is a human specific IgG1 anti-CD38 antibody, which has been approved as a single drug or combined regimen for the treatment of recurrent/refractory multiple myeloma. Daratumumab triggers CD38^+^ multiple myeloma cells [via antibody-dependent cellular cytotoxicity (ADCC), complement dependent cytotoxicity (CDC), and tumor-associated macrophages (TAMs)] in sensitive and drug-resistant patients, regulates the enzyme activity of CD38, reduces the level of adenosine and reduces adenosine-induced immunosuppression. In addition, Daratumumab reduces the types of inhibitory cells in the TME of multiple myeloma, i.e., it consumes immuno-suppressive cells such as CD38^+^ MDSCs, Tregs, and Bregs, and enhances anti-tumor activity (1). Moreover, in patients with a partial or good response to Daratumumab, cytotoxic T cells increased significantly and enhanced anti-tumor activity. Similarly, Isatuximab can also induce CD38^+^ Treg depletion and induce the proliferation and functional recovery of effector T cells (**Figure 2**). This suggests that Treg consumption is a key additional mechanism of action of these mAbs (38).

It was found that patients with at least a partial response to Daratumumab showed higher CD38 expression on MM cells (39). In this case, all-transretinoic acid (ATRA) treatment could increase the expression level of CD38 and reduce the expression of complement inhibitory proteins, CD55 and CD59, in MM cells, indicating that ATRA enhances the activity of Daratumumab (40). In addition, the use of immunomodulatory drugs (IMID), such as thalidomide, lenalidomide, and pomalidomide, resulted in the upregulation of CD38 on MM cells, causing them to trigger Daratumumab-induced NK cell-mediated ADCC (41, 42). Vitamin D can enhance cytotoxic activity *in vitro*; therefore, vitamin D supplementation in an IMID combined test could further improve the therapeutic effect of anti-CD38 antibodies (43). That study also found that the use of DNA methyltransferases (DNMTs) could achieve similar effects. Two DNMTs, azacitidine and decitabine, upregulated *CD38* mRNA levels in MM cells and the amount of CD38 on the cell surface. Therefore, the level of *in vitro* ADCC in cells treated with DNMT was higher than that of untreated cells, supporting the concept that DNMT can be used to improve the therapeutic effect of Daratumumab (44).

### Targeted CD38 Therapy for Melanoma

Primary melanoma cell lines can inhibit the proliferation of CD4^+^ and CD8^+^ T cells through adenosine-dependent mechanisms; however, it has been found that the use of CD38 inhibitors can reverse this effect and restore T cell proliferation (45). Therefore, blocking the CD38-mediated adenosine pathway seems to reduce the immunosuppression in melanoma (46). A study found that untreated control mice formed well vascularized tumors and developed lung metastasis compared with melanoma-infected mice treated with the NAADP inhibitor Ned-19, suggesting that targeted CD38 inhibition in melanoma is partly caused by reduced NAADP production (47).

### Relationship Between Targeted CD38 Therapy and Programmed Cell Death 1 (PD-1/PD-1 Ligand 1 (PD-L1) Efficacy

**Figure 3:**
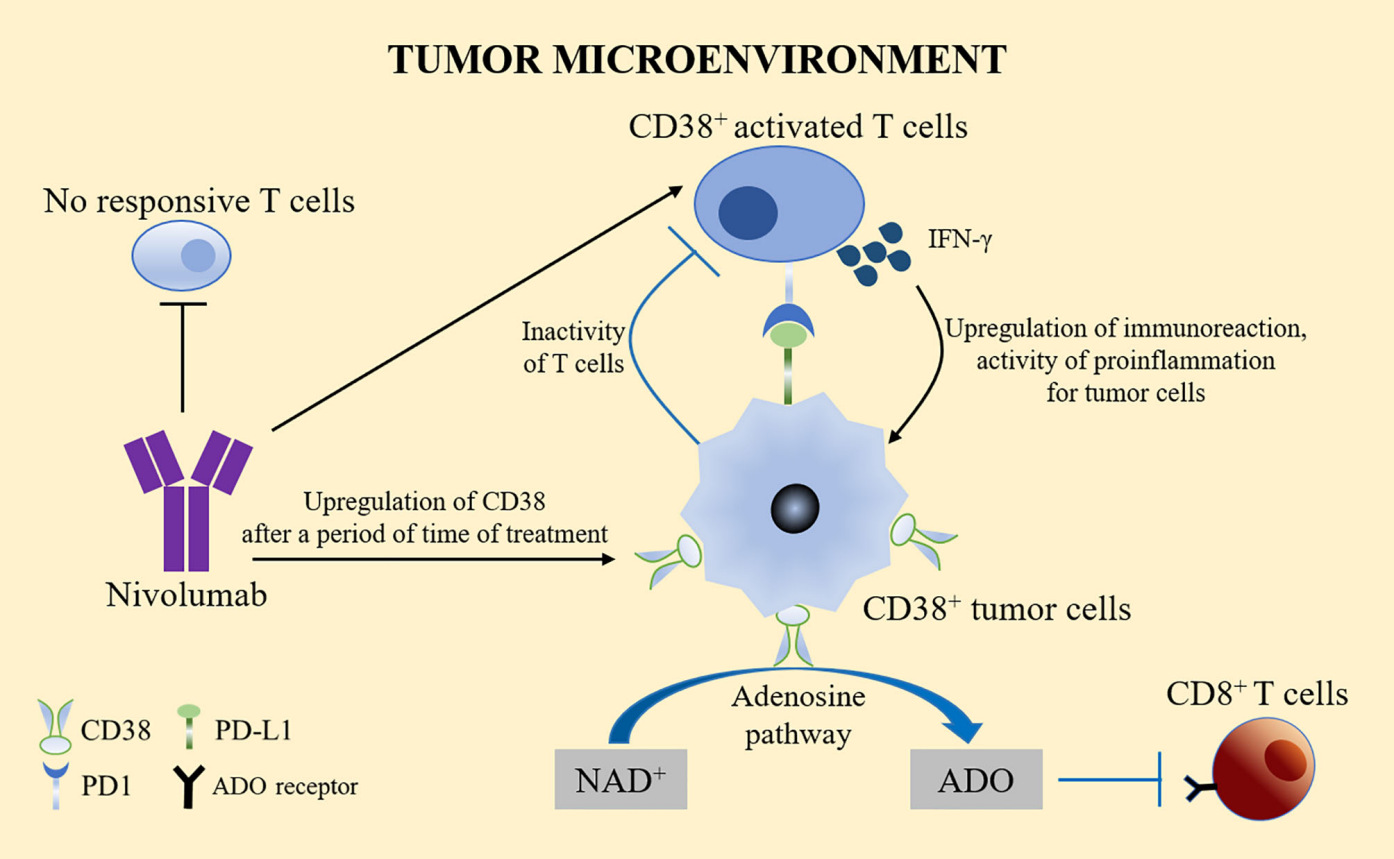
CD38 treatment and PD-1/PD-L1. Nivolumab significantly reduced the number of non-reactive T cells and increased the number of activated T cells expressing CD38. Activated CD38^+^ TILs produced cytotoxic compounds and inflammatory cytokines, such as IFN-γ to upregulate the immune response and exert pro-inflammatory effects on immune infiltration and tumor cells. However, CD38 is usually upregulated after a period of anti-PD-1/PD-L1 treatment. The adenosine activity of CD38 in turn leads to the inhibition of CD8^+^ T cells, which might partly explain the high drug resistance rate observed in patients treated with PD-1/PD-L1 blockers. Pharmacological targeting of the adenosine pathway could reverse the immunosuppression caused by the upregulation of CD38.

The use of the PD-1 specific monoclonal antibody nivolumab resulted in tumor volume reduction in one fifth of patients with advanced liver cancer. Nivolumab works by significantly reducing the number of non-reactive T cells and increasing the number of activated T cells expressing CD38 (48). These activated CD38^+^ tumor infiltrating lymphocytes (TILs) produce cytotoxic compounds and inflammatory cytokines to attack tumors. These cytokines include IFN-γ, which plays a key role in tumor control, upregulates the immune response, and exerts a pro-inflammatory activity on immune infiltration and tumor cells (49). However, CD38 is usually upregulated after a period of anti-PD-1/PD-L1 treatment. The adenosine-promoting activity of CD38 in turn leads to CD8^+^ T cell inhibition (50). This phenomenon might partly explain the high resistance rate observed in patients treated with PD-1/PD-L1 blockers, thus limiting the therapeutic benefits of anti-PD-1 immunotherapy. By inhibiting the adenosine receptor, pharmacological targeting of the adenosine pathway could reverse the immunosuppression caused by the upregulation of CD38 (**Figure 3**).

In another study, it was found that CD38 was the only significantly upregulated gene or protein after anti-PD-L1 treatment in patients with non-small cell carcinoma (NSCLC). Interferon-β and ATRA are important mediators for the upregulation of CD38 in NSCLC cells, which allows the tumor to develop resistance to anti-PD-L1 and anti-PD-1 treatments. This suggests that CD38 might be a potential additional immunotherapeutic target in HCC and NSCLC, and can be used together with PD-1/PD-L1 immunotherapy (51).

### Reasons for Choosing CD38 as a Therapeutic Target

**Figure 4:**
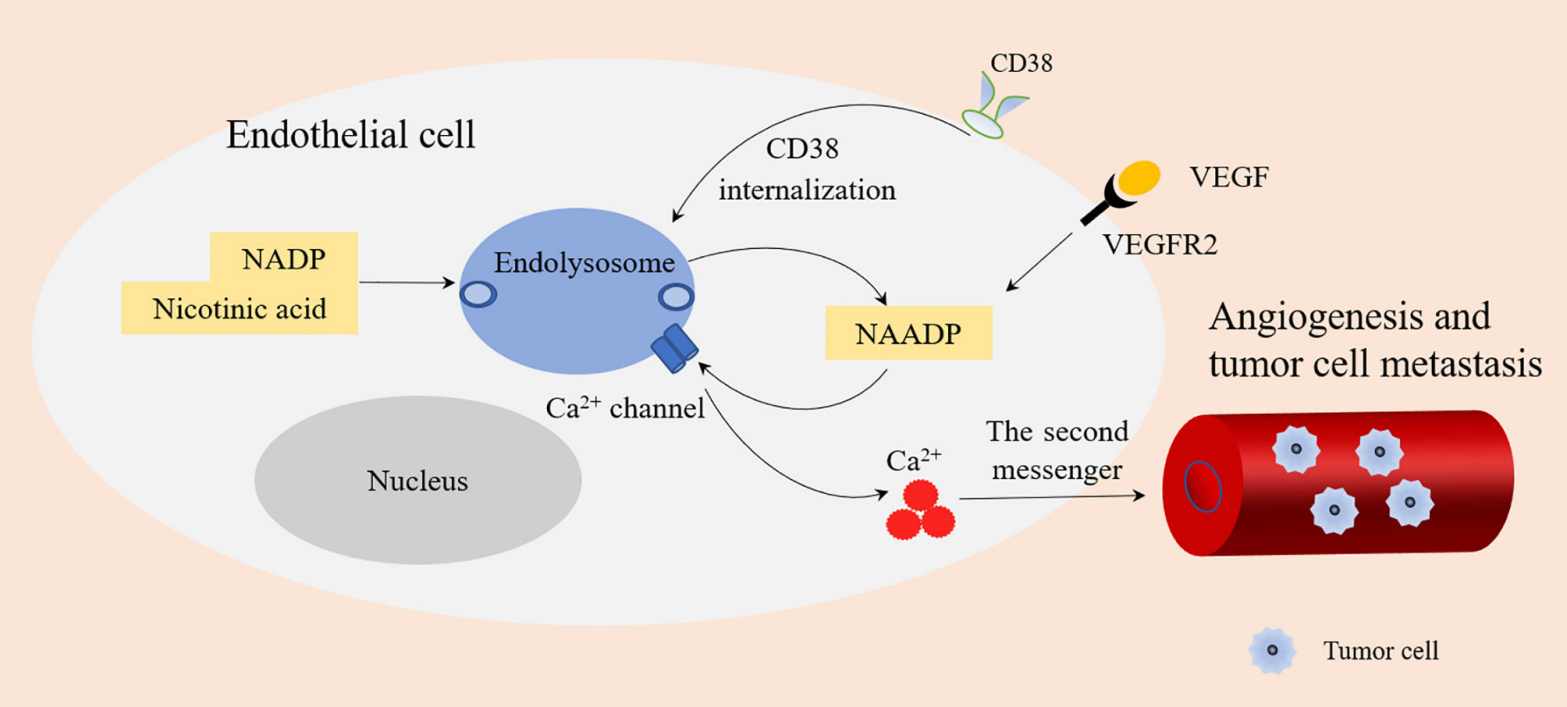
NAADP produced by CD38 and angiogenesis. Type II CD38 is expressed on the cell surface and internalized into endolysosomes through endocytosis. Endolysosomes provide an acidic environment where NADP and nicotinic acid are transformed into NAADP by base exchange reaction. NAADP acts on Ca^2+^ channels of Endolysosomes to promote the release of Ca^2+^ (as a second messenger), which is conducive to angiogenesis and tumor cell metastasis. VEGF binds with its receptors VEGFR2 to stimulate the release of Ca^2+^ by VEGFR2/NAADP/TPC2/Ca^2+^ signaling pathway leading to angiogenesis.

The main reasons why CD38 has become a tumor treatment target are as follows: (1) In a hypoxic TME, CD38 acts as an extracellular enzyme to catalyze NAD^+^ existing in the TME into ADPR or cADPR. This is the first step in another adenosine production pathway, which typically includes CD39 catalyzing adenosine triphosphate (ATP) to produce adenosine monophosphate (AMP). AMP is then generated from ADPR by CD203. Both pathways rely on CD73 to convert AMP into the final product, namely adenosine (52). Tumor cells can attract different regulatory subsets in the tumor microenvironment, such as Tregs, Bregs, CD16-CD56 bright NK cells, and MDSCs. All these cells may produce adenosine (ADO) from ATP or NAD^+^ (7). Under hypoxia, adenosine inhibits the anti-tumor immune response by recruiting bone marrow-derived suppressor cells and Tregs, thereby inhibiting the activity of T effector cells (36, 37). This leads to increased immune resistance of tumor cells and allows faster growth and proliferation rates (53). Therefore, the high expression of CD38 in the TME may lead to poor prognosis. (2) NAADP produced by CD38 is closely related to angiogenesis induced by vascular endothelial growth factor (VEGF) (54). CD38 is an ecto-enzyme highly expressed in endothelial cells (55). Type II CD38 can be internalized into endolysosomes through endocytosis. The acidic environment of endolysosomes is conducive to the production of NAADP (6, 56). NAADP stimulates Ca^2+^ channel of endolysosomes to releases Ca^2+^, and Ca^2+^ acts as the second messenger to promote angiogenesis, thus promoting tumor growth and metastasis (3). In addition, VEGF is the main angiogenic growth factor, which binds with its receptors VEGFR2 to stimulate the release of Ca^2+^ by VEGFR2/NAADP/TPC2/Ca^2+^ signaling pathway, which also leads to angiogenesis (57) (**Figure 4**).

Macrophages are the key mediators by which monoclonal antibodies to play a therapeutic role, and antibodies targeting CD38 will also affect macrophage function (45). Therefore, the role of CD38 in macrophage diseases, especially in tumor diseases, cannot be ignored. Therefore, targeted CD38 therapy would be a promising treatment for a variety of diseases.

## Summary and Prospects

CD38 has dual functions, acting as an extracellular enzyme and a surface receptor. As a double-edged sword, it ubiquitously expressed in immune cells such as T cells, NK cells, and dendritic cells. CD38 promotes the migration phenotype and signaling cascade, which is responsible for the activation and proliferation of various immune cells. Pro-inflammatory M1- like macrophages accumulate in metabolic tissues during aging and acute inflammatory reactions. The inflammatory cytokines secreted by aging cells (SASP) induce macrophage proliferation and enhance CD38-dependent NADase activity, thereby reducing the level of NAD in tissues. The decrease of NAD^+^ levels related to aging might weaken SASP and reduce its pathological effect. Macrophages might be the main reason for the increased expression of CD38 and decrease of NAD related to aging in aging tissues.

However, more experiments are needed to verify the following aspects: The role and possible mechanism of CD38 in macrophage polarization and proliferation; the exact relationship between CD38, macrophages, and injury to (and protection of) the nervous system; and the real relationship between targeted CD38 therapy and the efficacy of PD-1/PD-L1-targeted therapy.

## Author Contributions

WL and YL wrote the manuscript and drew the figures. XJ, QL and ZC collected the related papers and helped to revise the manuscript. HP and YZ designed and revised the manuscript. All the authors read and approved the final version of the review.

## Funding

This work was supported by the Science and Technology Foundation Survey Project of Ministry of Science and Technology of China (grant numbers 2018FY100900 and 2018FY10090004); the Hunan Provincial Natural Science Foundation (grant number 2021JJ30915); and the Fundamental Research Funds for the Central Universities of Central South University (for XJ).

## Conflict of Interest

The authors declare that the research was conducted in the absence of any commercial or financial relationships that could be construed as a potential conflict of interest.

## Publisher’s Note

All claims expressed in this article are solely those of the authors and do not necessarily represent those of their affiliated organizations, or those of the publisher, the editors and the reviewers. Any product that may be evaluated in this article, or claim that may be made by its manufacturer, is not guaranteed or endorsed by the publisher.
